# Micro RNA clusters in maternal plasma are associated with preterm birth and infant outcomes

**DOI:** 10.1371/journal.pone.0199029

**Published:** 2018-06-27

**Authors:** Joel C. Wommack, Jerome P. Trzeciakowski, Rajesh C. Miranda, Raymond P. Stowe, R. Jeanne Ruiz

**Affiliations:** 1 Microgen Laboratories, La Marque, TX, United States of America; 2 Texas A&M University, College of Medicine, Bryan, TX, United States of America; Gustave Roussy, FRANCE

## Abstract

The current study examined micro RNA (miRNAs) clusters from the maternal plasma to determine their association with preterm birth (PTB) and infant birth outcomes. A subsample of 42 participants who spontaneously delivered either preterm (≤37 weeks) or term was selected from a parent sample of 515 pregnant Mexican American women. Plasma samples and prenatal data were collected at a single mid-gestation time point (22–24 weeks’ gestation) and birth outcomes were obtained from medical records after delivery. Circulating miRNAs were analyzed by qPCR. When miRNAs were grouped according to chromosomal cluster rather than expression level, individual miRNAs correlated strongly with other individual miRNAs within their respective genomic locus. miRNAs from the c19mc cluster negatively correlated with c14mc miRNAs, and this relationship was more pronounced in PTB. Clusters c14mc was negatively associated with length of gestation; while the c19mc was positively associated with length of gestation and infant head circumference. Together, these findings suggest that groups of miRNAs from common chromosomal clusters, rather than individual miRNAs, operate as co-regulated groups of signaling molecules to coordinate length of gestation and infant outcomes. From this evidence, differences in cluster-wide expression of miRNAs are involved in spontaneous PTB.

## Introduction

Preterm birth (PTB), or gestational length ≤37 weeks, is probably the most significant obstetrical problem we currently face. Babies born too soon and too small often have multiple problems such as respiratory problems, intraventricular hemorrhages, necrotizing colitis and neurodevelopmental delays [1]. PTB is currently the primary reason for newborn mortality [2]. The primary monetary costs of PTB total to approximately $26 billion per year in the U.S. [1]. However, the secondary burdens of PTB may be even greater due to the lifelong health impact of premature birth [3]. Thus despite ongoing research, further understanding of the multifactorial etiologies that may affect PTB and related poor birth outcomes is still vitally needed. Scientists need to contemplate multiple risk factors and pathways leading to PTB—as well as their interactions [4]. One particular area of interest related to the etiology of PTB has recently been focused on epigenetics (for which microRNAs are a portion) [4,5]. The origins of PTB may be from complex gene-environment interactions. We present our results using a new methodology to examine microRNAs (miRNAs) as part of a pathway related to gestational age, PTB, and deleterious infant outcomes such as low birth weight and smaller head circumference.

miRNAs are small, non-coding RNAs that post-transcriptionally regulate gene expression that are implicated in numerous biological pathways and pathologies, including those of PTB [6]. A number of studies have focused on the involvement of miRNAs in specific medical conditions that significantly overlap with PTB, including, preeclampsia, intrauterine growth restriction, and fetal alcohol syndrome (FAS-D) [6–9]. Studies focusing specifically on PTB have identified individual miRNAs within the maternal circulation or within specific reproductive tissues such as placenta [5–7, 10]. While promising, the emerging literature on miRNA and pregnancy complications (including PTB) has also produced inconsistent results that can be attributed to differing tissues sources, quantitative methodologies and analytical strategies across studies and between research groups [7].

Another potential source of inconsistent results is due to the preponderance of studies that focus on the analysis of miRNAs as individual elements rather than functional groups. Throughout the genome, individual miRNAs are found as groups or “clusters” that are co-localized within distinct chromosomal loci. As such, miRNA clusters share common regulatory elements, that coordinate the synchronized expression of groups of individual miRNAs [11–12]. Moreover, miRNA clusters often share common target motifs that overlap across other functional gene networks. This enables clusters to influence other biological pathways through post-transcriptional regulation of not just one but several genes within a common network. As such, analytical approaches that represent miRNAs as functional groups of signaling molecules may provide results that reconcile the inconsistencies of previous studies.

Despite the ubiquitous involvement of miRNA in biological and disease pathways, a few miRNA clusters are selectively associated with pregnancy, pregnancy complications, and birth outcomes, most notably the c14mc, c19mc clusters [6,7]. miRNAs from the c14mc and c19mc clusters share a few important characteristics in that they are: 1) predominantly produced by the placenta, 2) detectable in the maternal circulation throughout gestation and 3) rapidly disappear from the maternal circulation following pregnancy termination [6–7, 13–15]. As demonstrated by clinical and *in vitro* studies, these clusters also show differing temporal expression patterns across pregnancy, wherein c14mc miRNA levels decrease from 1^st^ to 3^rd^ trimester and c19mc miRNA levels increase during this time [13, 16]. As circulating miRNA levels correspond to tissue expression [17], parallel c14mc and c19mc miRNA expression trajectories are likely present in the maternal plasma across pregnancy. Interestingly, c14mc and c19mc are the largest known miRNA clusters in the human genome, each consisting of 46 or more individual miRNAs located in distinct regions (40-100kb segments) on chromosomes 14 and 19, respectively [18–22]. Thereby, circulating c14mc and c19mc miRNAs potentially represent large functional groups of signaling molecules with selective involvement in pregnancy.

Indeed, empirical studies on plasma samples collected during mid-gestation have demonstrated the involvement of individual miRNAs from the c14mc and c19mc clusters in pregnancy outcomes and complications such as PTB or preeclampsia [7]. These effects were observed in plasma samples collected during the 1^st^ trimester [23] as well as mid-gestation [5]. Mid- and late-gestation sampling and analysis of groups circulating miRNAs that included individual members of c14mc and c19mc clusters has also demonstrated altered expression in pregnancies resulting in Fetal Alcohol Syndrome, a behavioral health condition that considerably overlaps with PTB [9].

As, investigations into circulating miRNA and prenatal health have focused primarily on individual miRNAs rather than clusters of miRNAs and have often investigated pathologies that overlap PTB, we propose that cluster-level analysis in pregnancies resulting in spontaneous PTB (without major coinciding complications) will more clearly characterize relationships between circulating miRNA, PTB, and associated infant outcomes. Therefore, the current study sought to 1) investigate cluster-wide associations of pregnancy specific miRNA with length of gestation and birth outcomes and 2) examine whether differences in coordinated expression of circulating miRNA were associated with PTB.

## Methods

The current study was conducted in a subset of a parent study (n = 515) on pregnant Hispanic women recruited at two obstetrical clinics in the Houston metropolitan areas from 2008–2012 [24]. The subsample (n = 42) consisted of patients who spontaneously delivered before the completion of 37 gestational weeks (PTB; n = 21) and patients who spontaneously delivered after 37 weeks (Term; n = 21). Selected cases of PTB and term pregnancy were matched for age, gravidity, and BMI. Enrollment criteria included patients who: were between the ages 19 and 39; self-identified as Hispanic; had the ability to read and speak English and/or Spanish; were born in the U.S. or had lived in the U.S. for 10 or more years; were covered by self-pay, state funded or private insurance. Exclusion criteria included: pregnancies with twins or multiple fetuses; medical risk factors such as chronic hypertension, type I or II diabetes, gestational diabetes with insulin treatment, thyroid disorder; major psychiatric disorders (e.g. Bipolar Disorder, Schizophrenia); use of steroids or antidepressants one month prior to enrollment; fetal or uterine anomalies as determined by ultrasound; fetal demise; placenta previa, preeclampsia at time of data collection; cerclage.

Data were collected between 22–24 weeks’ gestation during an appointment with a research nurse separately scheduled from regular prenatal care visits. Venipuncture blood draws (20ml) were collected by between 2 and 4 pm to control for diurnal variation. Immediately following blood collection, all samples were centrifuged and plasma was separated and aliquoted for storage at -80°. Prenatal data was collected from prenatal records for age, gravidity, and BMI and history of PTB. Protocols for this study were approved by the institutional review boards at The University of Texas Medical Branch, Galveston, TX and The University of Texas at Austin.

### miRNA analysis

Plasma samples were thawed and centrifuged at 3000 x g for 5 minutes. For each sample, a 200μl aliquot was transferred to a microtube and combined with a lysis buffer containing 1μg carrier RNA per 60μl buffer. Total RNA was extracted using miRCury RNA isolation kit (Exiqon, Woburn, MA). Each miRNA sample was reverse transcribed into cDNA and run on miRCURY LNA^TM^ Universal RT microRNA PCR Human Panels 1&2 (Exiqon, Woburn, MA). Raw CT values were subject to quality control checks for hemolysis [9]. Cycle thresholds (CTs) were considered un-amplified if CT values > 50. -∆CTs for each individual miRNA in each sample was normalized to the mean CT for all miRNAs in that particular sample (e.g. globalized miRNA expression) and calculated as (CT_miRNA_x_-CT_Average_miRNA_Expression_). In a previous study, we demonstrated that globalized miRNA expression within maternal plasma is unaffected by experimental condition [9].

### miRNA clusters

miRNA clusters were classified as groups of individual miRNAs within 10kb on the same chromosome via mirbase.org [25]. Specifically, miRNAs from the c14mc cluster were defined as individual miRNA located at position 14q32. on chromosome 14, and c19mc miRNAs were selected from position 19q13.41 on chromosome 19. miRNAs from the family cluster miR-17/92 were located within a 10kb proximity on chromosome 13, position 13q31.1 and another group of these miRNA were chromosomally co-localized on the X chromosome, position Xq26.2 [26]. For inclusion in statistical analysis, individual miRNAs had to be detected in a minimum of 30/42 patient samples; miRNAs that fell below this threshold were excluded from the present analyses. A list of each individual miRNA used for cluster variables is provided in S1 Table.

### Statistical analysis

Initial analysis compared individual miRNAs expressed as -∆CTs between term and PTB pregnancies by t-test. Additionally, correlational analysis for individual miRNAs within and between chromosomal clusters was conducted using Pearson’s.

To examine potential differences in cluster-wide expression patterns between term and PTB samples we compared correlation matrices of individual miRNA expression levels grouped by genomic location. For each chromosomal cluster, we created a correlation matrix of the -∆CTs between each miRNA within that particular genomic location using values from only term or only PTB samples. Correlation coefficients from term or PTB samples were compared statistically by the Jennrich test [27], which tests the equality of two matrices. As the sets of coefficients followed normal distributions, we also used one-way ANOVA with pairwise comparisons to determine if a significant difference existed between the two groups.

Factor analysis using minimum residual exploratory factor analysis (EFA) was performed in R (R Core Team (2016). R: A language and environment for statistical computing. R Foundation for Statistical Computing, Vienna, Austria. URL https://www.R-project.org/)., using the psych package (Revelle, W. (2017) psych: Procedures for Personality and Psychological Research, Northwestern University, Evanston, Illinois, USA, https://CRAN.R-project.org/package=psych Version = 1.7.5). The number of dimensions to extract was determined by examining Bayesian Information Criteria (BIC) and with parallel analysis, which compares the observed eigenvalues of the correlation matrix with those from simulated data with similar properties to the actual data [28]. The factor solution was rotated into an oblique solution using the oblimin transformation to simplify interpretation while preserving correlation structure (S2 Table). Factor variables were constructed by row means for each miRNA from a respective cluster. miRNA Factor variables were then used to assess differences in gestational age at birth between term and preterm deliveries. Each matched pair of women was assigned an ID number which was used as a random factor in linear or logistic mixed regression models. Fixed factors in these models included gestational age at birth, and miRNA cluster variables. Additionally, fetal sex, history of PTB, gravida, and pre-pregnancy BMI, factors known to pregnancy outcomes, were included as covariates. Results were considered significant if the p-value for the interaction between the acculturation measure and cluster variable were below 0.05.

Each t-test, ANOVA, linear mixed-effects model, and Pearson’s correlation was assessed with the significance level at 0.05. Data were analyzed using Stata 14 Software (StataCorp 2015. Stata Statistical Software: Release 14. College Station, TX: StataCorp LP).

## Results

### Sample characteristics

Table 1 illustrates that differences in length of gestation between term and PTB cases were unlikely due to medical complications (e.g. preeclampsia) that are often factors for PTB. Additionally, term and PTB cases did not significantly differ in demographic characteristics (e.g. age, pre-pregnancy BMI, income). While the parent sample included patients from Houston and Austin, the subsample selected for this study only included patients from the Houston area to avoid confounding factors possibly associated with geographic region. Patients who had a history of PTB were in both the term and PTB groups, however, none of these patients received progesterone treatment during their prenatal care.

**Table 1:** Sample characteristics (frequency or mean ± SD).

	Total (n = 42)	Term (n = 21)	Preterm (n = 21)
**Demographics**			
Age	26.25 ± 5.05	26.3 ± 4.8	26.2 ± 5.3
Pre-Pregnancy BMI	25.7 ± 5.35	25.8 ± 5.5	25.6 ± 5.2
Cotinine Positive	2	1	1
**Pregnancy Outcomes**			
Spontaneous Delivery	42	21	21
Gestational Age at Birth (Weeks)	36.9 ± 1.6	38.6 ± 0.7	35.3 ± 2.4
Birthweight (g)	2852 ± 410.1	3114 ± 286.5	2590 ± 533.6
Infant Head Circumference (cm)	32.6±1.8	33.1±1.2	32.1±2.2
Preeclampsia	0	0	0
Chorioamnionitis	0	0	0
Preterm PROM	0	0	0
Diabetes (GDM or PGDM)	0	0	0
Placental Abruption	0	0	0
**Maternal History**			
Gravida			
* 1 Pregnancy*	7	3	4
* 2 Pregnancies*	7	3	4
* 3 Pregnancies*	16	10	6
* 4+ Pregnancies*	12	5	7
History of PTB	10	3	7
Progesterone Treatment	0	0	0

### miRNA

#### Individual expression

Initial comparisons of individual miRNA expression showed differences between term and PTB compared by t-test (S1 Table) for three miRNAs from c14mc: miRNAs hsa-mir-127-3p and hsa-mir-136-5p from the RTL subregion of c14mc and hsa-mir-543 located in a broader region of the c14mc cluster. No other group differences were observed between term and PTB samples for any other individual miRNAs from the c14mc cluster; nor were any group difference observed between individual miRNAs from the c19mc or miR-17/92 cluster. Total miRNA expression did not differ between term and PTB samples [t(39) = 0.09; p = 0.9]

#### Expression by chromosomal clusters

**Fig 1:**
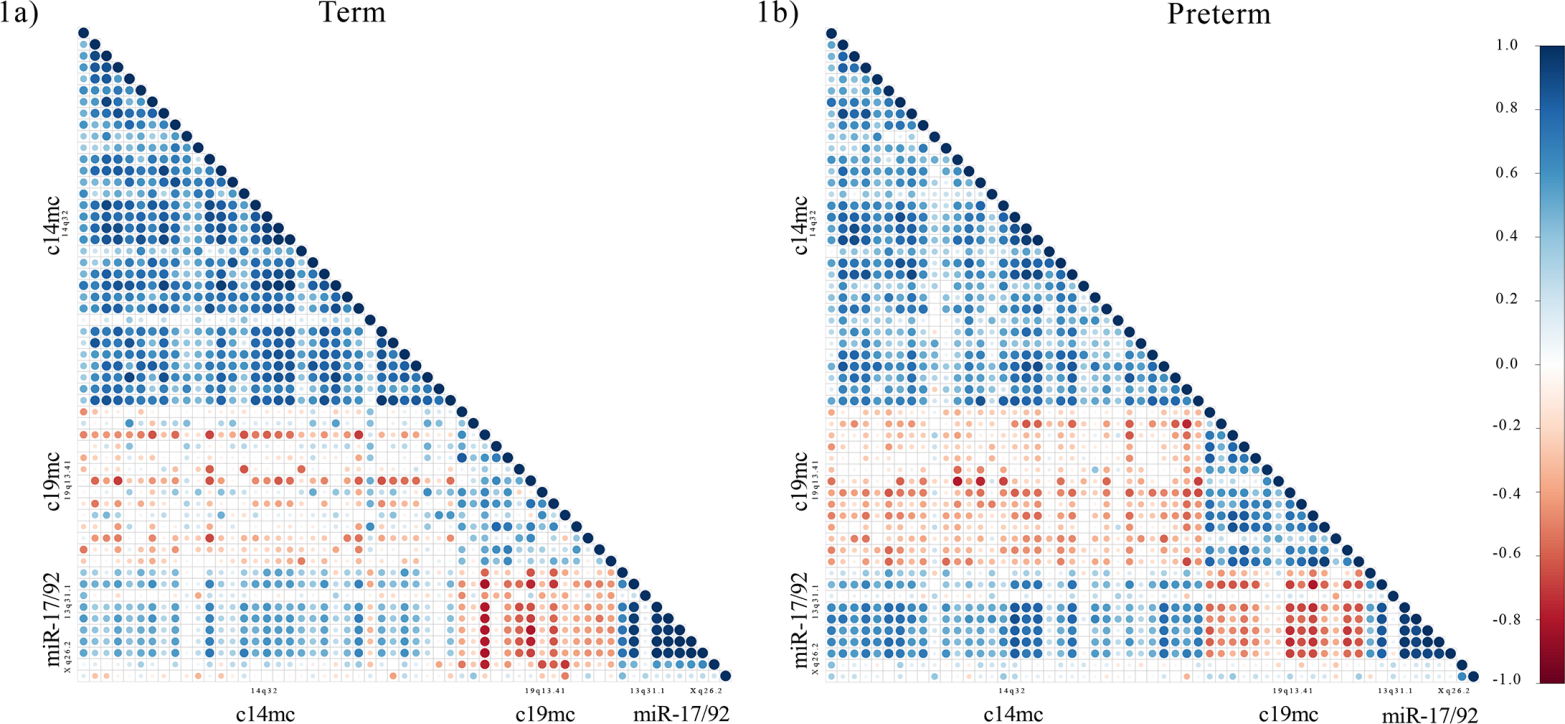
Correlations plots of individual miRNAs (-∆CTs) from clusters c14mc, c19mc, and miR-17/92. Individual miRNAs were grouped according to cluster and ordered within according to genomic position clusters. Pearson’s correlation analysis was performed for individual miRNAs -∆CTs for term (A) and preterm (B) separately. Positive and negative Pearson’s r coefficients are represented by blue and red dots, respectively.

To examine the relationship between expression levels of miRNAs within chromosomal clusters and between miRNAs from separate genomic loci, we performed correlational analysis using Pearson’s correlation. Consistently positive correlations were observed between miRNAs and the other miRNAs from the same chromosomal clusters. Negative correlations were observed in expression levels between c19mc miRNA and miRNAs from all other clusters (c14mc, and miR-17/92 located on chromosome 13 and the X chromosome). The positive correlations of miRNA with other miRNAs from the same chromosomal cluster is most apparent when a correlation matrix was organized according to genomic location (Fig 1). A correlation matrix that organized miRNA expression levels from highest to lowest, regardless, of genomic location, revealed no specific pattern of expression.

**Fig 2:**
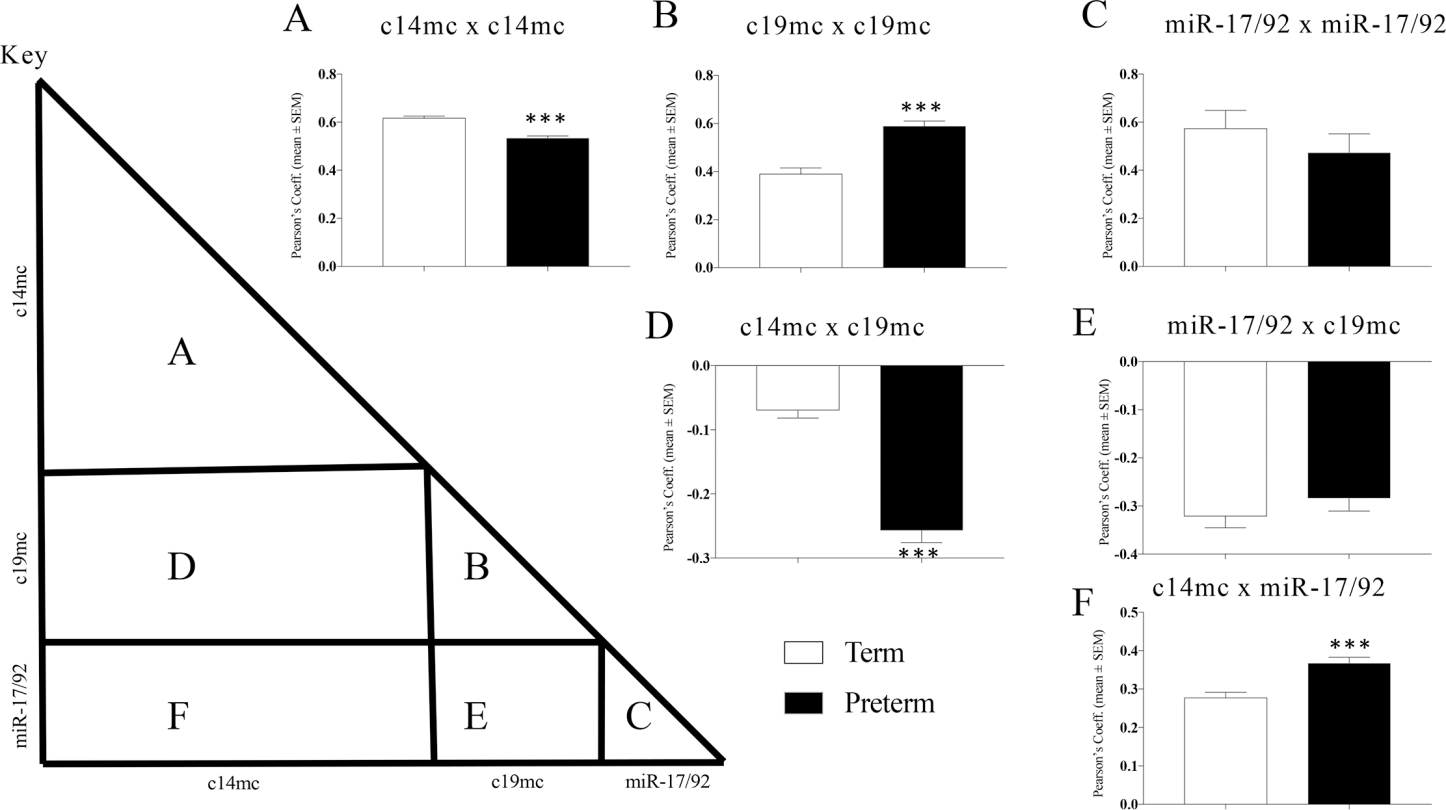
miRNA correlation comparisons between PTB and term cases. Average correlations between individual miRNAs were compared between term and PTB to evaluate differences in coordinated expression of miRNA clusters c14mc, c19mc, and miR-17/92. Regions of each respective term and PTB correlation matrices that corresponded to within cluster correlations (A-C) and between clusters correlations (D-F) were compared by ANOVA (Fig 2 key provides a visual guide for which areas of the matrices were compared between term and PTB). (A) PTB showed lower correlated expression of c14mc miRNAs and (B) higher correlated expression of c19mc miRNAs. (C) Within cluster correlations for miR-17/92 miRNAs did not differ between term and PTB. (D) Negative correlations between c14mc and c19mc miRNAs was stronger in PTB, while (E) the negative correlations between miR-17/92 and c19mc miRNAs did not differ between term and PTB. (F) PTB showed stronger correlations between c14mc and miR-17/92 miRNAs. Correlations were calculated using Pearson’s correlation coefficient. *** = p<0.001; ANOVA.

To further investigate patterns of co-expression, correlation matrices were created using either samples only from term (Fig 1A) or PTB (Fig 1B) deliveries. For within cluster correlations, c14mc miRNA were significantly more positively correlated with each other in plasma collected in term pregnancies than in PTB samples [F(1,1188) = 45.96; p<0.001] (Fig 2A). Co-expression of c19mc miRNAs with each other was significantly more positively correlated in PTB samples than in term samples [F(1,208) = 37.83; p<0.001] (Fig 2B). We observed no differences in the average correlation between miR-17/92 between term and PTB samples [F(1,88) = 2.95; p = 0.09] (Fig 2C). For between cluster comparisons, the mean negative correlation between c14mc and c19mc miRNA was significantly greater in PTB as compared to term samples [F(1,1019) = 160.24; p<0.001] (Fig 2D). Correlations between c19mc and miR-17/92 miRNAs did not differ between term and PTB [F(1,310) = 1.12; p = 0.29] (Fig 2E). However, higher correlations between c14mc miRNAs and miR-17/92 miRNAs located were detected in PTB samples [F(1,678) = 19.22; p>0.001] (Fig 2F).

### Factor analysis and miRNA cluster variables

Correlation plots using all miRNAs from c14mc, c19mc and miR-17/92 for each patient (Term or PTB) indicated that miRNA expression was coordinated by genomic location (Fig 1). We also observed consistent positive correlations between individual miRNAs from the c14mc and miR-17/92 clusters and birth outcomes (length of pregnancy, birth weight, and infant head circumference) (Table 2). Similarly, we observed consistent negative correlations between c19mc miRNAs and birth outcomes, indicating that high c19mc expression is related to pregnancies that are longer in duration as well as greater infant birth weight head circumference. A separate correlation matrix that ranked miRNA from high to low expression, regardless of genomic location, showed no distinct patterns (S1 Fig). In this correlation plot, individual c14mc miRNAs that were more highly expressed in PTB samples (hsa-mir-127-3p, hsa-mir-136-5p, and hsa-mir-543) were randomly interspersed between miRNAs from clusters c19mc and miR-17/92. A correlation matrix consisting of miRNA grouped by expression rank rather than genomic location revealed no cohesive pattern or relationship with any other birth outcomes.

**Table 2:** Correlations between individual miRNAs birth outcomes.

miRNA	Cluster	Pregnancy Duration	Birth Weight	Infant Head Size
hsamir4935p	c14mc	0.1494	0.1916	0.3751*
hsamir3373p	c14mc	0.3281*	0.3486*	0.2178
hsamir3375p	c14mc	0.1995	0.2899	0.1657
hsamir4315p	c14mc	0.2927	0.31*	0.2913
hsamir4333p	c14mc	0.2480	0.1654	0.1561
hsamir1273p	c14mc	0.471*	0.3522*	0.3159
hsamir4325p	c14mc	0.2294	0.2739	0.3748*
hsamir1363p	c14mc	0.2975	0.3458*	0.1760
hsamir1365p	c14mc	0.4058*	0.3574*	0.2689
hsamir3793p	c14mc	0.3024	0.3190	0.1204
hsamir3795p	c14mc	0.1462	0.1145	0.2870
hsamir3803p	c14mc	0.3409	0.4181*	0.4425*
hsamir323a3p	c14mc	0.323*	0.3335*	0.1944
hsamir3293p	c14mc	0.1518	0.1969	0.0764
hsamir4943p	c14mc	0.1201	0.2690	0.3952*
hsamir543	c14mc	0.3476*	0.379*	0.2961
hsamir4953p	c14mc	0.3496*	0.2913	0.3197*
hsamir376c3p	c14mc	0.3074*	0.2860	0.3002
hsamir376a3p	c14mc	0.2851	0.2930	0.2769
hsamir376a5p	c14mc	0.3339*	0.2600	0.1940
hsamir6543p	c14mc	0.2114	0.2860	0.2169
hsamir376b3p	c14mc	0.2694	0.2561	0.2458
hsamir3813p	c14mc	0.1016	0.2328	0.0182
hsamir487b3p	c14mc	0.2511	0.2374	0.2072
hsamir8893p	c14mc	0.2071	0.2439	0.3329
hsamir3823p	c14mc	0.1295	0.2353	0.1619
hsamir1345p	c14mc	0.2274	0.2117	0.2027
hsamir496	c14mc	**0.3629**	0.2826	0.1404
hsamir3773p	c14mc	0.2303	0.2813	0.1999
hsamir4093p	c14mc	0.2637	0.2649	0.2713
hsamir3693p	c14mc	0.2028	0.2489	0.2080
hsamir3695p	c14mc	0.2608	**0.3352**	0.1564
hsamir4103p	c14mc	0.2520	0.1924	0.1176
hsamir5153p	c19mc	-0.0583	-0.0541	-0.2190
hsamir5155p	c19mc	-0.1079	-0.0454	-0.1140
hsamir520a5p	c19mc	-0.1380	-0.2998	-0.4409*
hsamir5255p	c19mc	-0.0995	-0.1893	-0.3597*
hsamir518f5p	c19mc	-0.2525	-0.2322	-0.3668*
hsamir520c3p	c19mc	-0.1809	-0.0622	-0.2170
hsamir518c3p	c19mc	-0.0860	-0.0833	-0.2413
hsamir518c5p	c19mc	-0.0499	-0.1059	-0.2282
hsamir5243p	c19mc	-0.3067	-0.2615	-0.5219**
hsamir5245p	c19mc	-0.2000	-0.2513	-0.3533*
hsamir517a3p	c19mc	-0.0828	-0.1044	-0.2788
hsamir519d3p	c19mc	-0.1789	-0.1978	-0.1910
hsamir518d5p	c19mc	-0.1760	-0.1988	-0.4283**
hsamir519a3p	c19mc	-0.1913	-0.2221	-0.4493**
hsamir173p	miR-17/92	-0.1043	-0.1378	0.0817
hsamir175p	miR-17/92	0.0675	0.0655	0.2355
hsamir18a3p	miR-17/92	-0.0933	-0.1150	0.0010
hsamir18a5p	miR-17/92	0.1267	0.0728	0.341*
hsamir19a3p	miR-17/92	0.1181	0.1077	0.3347*
hsamir20a5p	miR-17/92	0.0769	0.0180	0.3375*
hsamir106a5p	miR-17/92	0.1036	0.0360	0.348*
hsamir18b5p	miR-17/92	0.1591	0.0931	0.3647*
hsamir20b5p	miR-17/92	0.0091	0.0846	0.2117
hsamir3633p	miR-17/92	-0.0637	-0.0048	0.1495

Pearson's Correlation

* = p<0.05

** = p<0.01

Factor analysis confirmed observation miRNAs are better grouped by cluster than by up- or down-regulation, as individual miRNAs loaded on to latent variables that corresponded to genomic location rather than expression level (S2 Table). Individual miRNAs from c14mc, c19mc, and miR-17/92 loaded onto factors consisting of primarily miRNAs from the same respective cluster. We then created three factor variables for miRNA clusters using row means of the -∆CT for individual miRNAs within a respective chromosomal cluster: c14mc c19mc and miR-17/92. This approach accounted for any potential missing values resulting from miRNAs that were undetectable in a given patient’s sample. For c14mc, a cluster variable was created that consisted of each miRNA from c14mc, genomic location 14q32. For c19mc, a whole cluster variable was created that included all miRNAs from c19mc, genomic location 19q13.41. For the miR-17/92, a variable was created that included all miRNAs from this sequence-based cluster either 13q31.3 or Xq26.2 [26].

We then tested the ability of each factor variable cluster of miRNAs for association with birth outcomes (length of gestation, birth weight, and infant head circumference) in mixed model regressions with paired cases set as a random factor (Results summarized in Table 3). We controlled for pre-pregnancy BMI, gravida, history of PTB, and fetal sex by using these variables as covariates in each regression model. The c14mc cluster positively predicted length of gestation at birth with positive regression coefficients. Because miRNA expression was expressed as -∆CT, the positive coefficient indicates that high c14mc expression is predictive of early spontaneous delivery and is consistent with correlational analysis. Regression models using each of the miR-17/92 and c19mc clusters variables were not statistically significant. The correlation coefficient between c19mc and length of gestation was negative, suggesting that low expression of individual c19mc miRNAs is associated with PTB. For infant birth outcomes, we did not find a significant relationship between the c14mc or miR-17/92 clusters with either birth weight or infant head circumference. A positive relationship between c19mc and infant head circumference, however, was detected. An additional linear regression model using only samples from term deliveries showed that the relationship between c19mc and head circumference was independent in differences in length of gestation or infant birthweight (R^2^ = 0.584, F(5,14) = 3.93, p<0.05).

**Table 3:** Summary Relationships between miRNA clusters, length of Gestation and Infant Outcomes.

Mixed Model Regression for miRNA Clusters and Birth Outcomes					
Outcome	miRNA Cluster	Estimate	Std. Err	z	95% C.I.
**Length of Gestation**	c14mc	0.83*	0.39	2.11	0.06–1.60
	c19mc	-0.66	0.4	-1.64	-1.30–0.13
	miR-17/92	0.79	0.69	1.16	-0.55–2.15
**Infant Birthweight**	c14mc	189.21	86.25	2.19	20.16–358.25
	c19mc	-144.46	88.07	-1.64	-317.07–28.15
	miR-17/92	106.25	153.73	0.69	-195.05–407.55
**Infant Head Circumference**	c14mc	0.62	0.32	1.99	0.008–1.25
	c19mc	-0.87*	0.29	-3.05	-1.43–-0.31
	miR-17/92	1.14	0.52	2.21	0.127–2.16

Cluster variables based on chromosomal location were tested as predictors of birth outcomes (pregnancy duration, infant birth weight, and infant head circumference) by mixed model regression with pre-pregnancy BMI, infant sex, gravida, and history of PTB as control covariates. Statistical significance * = p<0.05 (bold).

## Discussion

The first major finding of our current study is that pregnancy specific miRNAs within the maternal plasma are co-regulated based on chromosomal clustering at distinct genomic loci. Previous studies into PTB and other prenatal medical complications that focused on individual miRNAs or groups of miRNAs sorted by expression levels rather than genomic location have produced disparate results. Findings from the current study provide a novel perspective by examining cluster-wide associations between circulating miRNAs and birth outcomes. Still, our results are consistent with previous studies that have identified individual miRNAs or groups of miRNAs from the c14mc, c19mc, and miR-17/92 chromosomal clusters that are associated with pregnancies disorders, including PTB [5], pre-eclampsia [8, 23], and FAS-D [9].

The second major finding from our study is that differential expression of miRNA clusters c14mc and c19mc are predictive of PTB. Both correlational analysis and mixed model, multivariate regressions demonstrated that pregnancy duration is predicted by c14mc and c19mc miRNA expression patterns. In both term and PTB pregnancies, c14mc and c19mc expression are inversely related. This negative correlation between these miRNA clusters is most pronounced in pregnancies that resulted in spontaneous PTB. As such, these findings indicate the difference in pregnancies resulting in term or PTB may be related to an imbalance in c19mc and c14mc expression. It is important to note, that none of the patients in the current study developed preeclampsia or gestational hypertension or gave birth to infants with symptoms of FAS-D. As such, differential expression of c19mc miRNAs in our study is most likely due to decreased pregnancy duration, and not a result of overlapping pathologies.

The results that various clusters of miRNAs analyzed here are highly related to the infant birth outcomes of birth weight and head circumference is important. Several of the miRNAs are highly related to head circumference, especially in the c19mc cluster. These findings are not necessarily in concert with birth weight and gestational length, which is intriguing. This is potentially an important finding, considering that the relationship between c19mc miRNAs and head circumference is independent of length of gestation and is definitely worthy of follow up with the infants to see if this is then related to their future neurodevelopment.

In the current study, we included clinically-based observations known to influence prematurity (history of PTB, gravida, BMI and fetal sex) as covariates in regression models analyzing the relationship between miRNA clusters and PTB. Importantly, none of these clinical observations significantly influenced the statistical models used to analyze the relationship between c14mc and c19mc clusters and length of gestation. Further investigations are needed to address potential relationships between circulating miRNAs and previously identified biological and/or sociocultural risk factors for PTB.

The c14mc and c19mc miRNAs are groups of biomarkers that share several key characteristics. Due to genomic co-localization, c14mc and c19mc miRNAs share regulatory mechanisms intrinsic to their respective cluster. Consequently, these common regulatory elements may selectively influence the conditions under which c14mc and c19mc miRNAs are expressed as groups, specifically where (e.g. tissue or cell type) and when (e.g. developmental period). For example, both clusters are preferentially produced within the placenta and thus most abundantly present in the circulation during pregnancy [13–15], and both clusters are subject to genomic imprinting, as c14mc miRNAs are expressed exclusively from the maternal allele while c19mc miRNAs are solely expressed from the paternal allele [6]. Moreover, miRNAs from c14mc and c19mc may also act as coherent functional groups through shared and/or overlapping target motifs. Bioinformatics resources (Qiagen Ingenuity Pathway analysis and Targestscan.org) indicate that several members of c14mc target genes involved in estrogen receptor signaling and several members of the c19mc cluster target genes regulating the biosynthesis of steroid hormones including estrogen, progesterone, and cortisol [29, 30]. As such, coordinated expression of c14mc and c19mc miRNAs may serve to refine expression of genes within selective canonical pathways involved in pregnancy. Further studies will be required to determine if and how these biomarkers act as groups to influence known pathways of PTB or other adverse pregnancy outcomes.

The findings from this study demonstrate that a different methodology of analyzing miRNAs may prove to be very beneficial in understanding the pathway(s) leading to PTB and associated infant outcomes. This methodology indicates that the clusters of miRNAs (particularly c14mc and c19mc) may function in groups physiologically, and therefore the disparate results from other studies [5] may be explained by differences in the methodology by grouping miRNAs by expression rather than cluster. The strength of the reported study is that it was a case-controlled sample that only had spontaneous PTB and did not have mixed outcomes, such as early delivery due to preeclampsia. It also had a controlled sample as to ethnicity, all being Mexican Americans, without medical complications putting them at high risk. Another strength of the work is that the miRNAs were obtained from plasma samples.

Future work in patient populations with different ethnicities and sociocultural backgrounds will be important for assessing the generalizability of the present findings. In addition, a longitudinal study is needed to help understand differences in how the clusters of miRNA work over time in the pregnancy, both for term and PTBs. A larger sample would also be of benefit to confirm the results reported here and further explore how miRNA clusters are related to PTB risk factors such as history of PTB.

The study of circulating miRNAs as biomarkers for PTB is relatively nascent. Thus far, studies on miRNA and PTB widely vary in terms of experimental design, tissue source, method of quantification and interpretation of findings. As a result, the collective findings are inconsistent across studies [7]. Thus, we emphasize the need for further investigations to clarify relationships between circulating miRNAs and adverse pregnancy outcomes.

## Supporting information

S1 Fig Correlation matrix ordered by expression rank. (A) Correlation matrix of individual miRNAs from c14mc, c19mc, and miR-17/92 clusters grouped ordered according to expression (highest to lowest -∆CT) rather than cluster or genomic location. Rows for correlations for select individual miRNAs from the c14mc and c19mc cluster are highlighted in yellow. (B) Correlations between individual miRNAs (ranked by -∆CT) and birth outcomes: length of gestation, birth weight, and infant head circumference. Displayed correlation matrix was analyzed using the whole sample of 42 patients who delivered spontaneous either term (n = 21) or preterm (n = 21). Positive and negative Pearson’s r coefficients are represented by blue and red dots, respectively.
(TIFF)

S1 Table Expression of individual miRNAs for PTB and term cases. (XLSX)

S2 Table Factor loading coefficients for each individual miRNA. (XLSX)
