# Supplementary figures and images for: Micro RNA clusters in maternal plasma are associated with preterm birth and infant outcomes
Source: PLoS One. 2018 Jun 27;13(6):e0199029. doi: 10.1371/journal.pone.0199029 (PMC6021076; doi:10.1371/journal.pone.0199029)

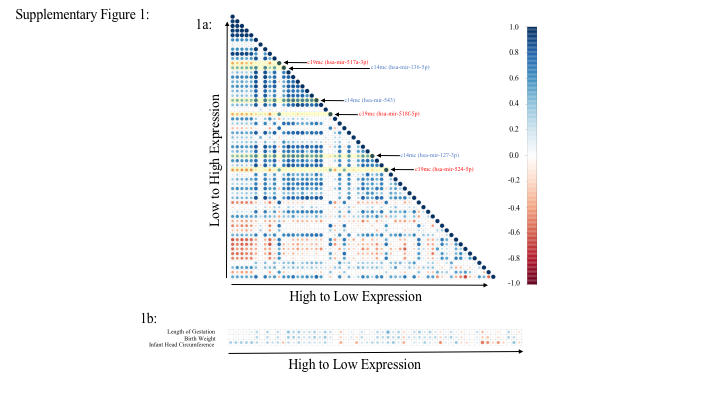

Supplement: S1 Fig — (A) Correlation matrix of individual miRNAs from c14mc, c19mc, and miR-17/92 clusters grouped ordered according to expression (highest to lowest -∆CT) rather than cluster or genomic location. Rows for correlations for select individual miRNAs from the c14mc and c19mc cluster are highlighted in yellow. (B) Correlations between individual miRNAs (ranked by -∆CT) and birth outcomes: length of gestation, birth weight, and infant head circumference. Displayed correlation matrix was analyzed using the whole sample of 42 patients who delivered spontaneous either term (n = 21) or preterm (n = 21). Positive and negative Pearson’s r coefficients are represented by blue and red dots, respectively. (TIFF) [file pone.0199029.s001.TIFF]
